# Polysomnography Is an Important Method for Diagnosing Pediatric Sleep Problems: Experience of One Children’s Hospital

**DOI:** 10.3390/children8110991

**Published:** 2021-11-02

**Authors:** Chien-Heng Lin, Chieh-Ho Chen, Syuan-Yu Hong, I-Ching Chou, Shinn-Jye Liang, Liang-Wen Hang

**Affiliations:** 1 Division of Pediatric Pulmonology, China Medical University Children’s Hospital, Taichung 404327, Taiwan; lch227@ms39.hinet.net (C.-H.L.); d30270@mail.cmuh.org.tw (C.-H.C.); 2 College of Medicine, China Medical University, Taichung 404328, Taiwan; dazingdog@hotmail.com; 3 Division of Pediatric Neurology, China Medical University Children’s Hospital, Taichung 404327, Taiwan; iching@mail.cmu.edu.tw; 4 Graduate Institute of Integrated Medicine, China Medical University, Taichung 404328, Taiwan; 5 Sleep Medicine Center, Department of Pulmonary and Critical Care Medicine, China Medical University Hospital, Taichung 404327, Taiwan; d6142@mail.cmuh.org.tw; 6 School of Nursing & Graduate Institute of Nursing, China Medical University, Taichung 404328, Taiwan

**Keywords:** polysomnography, obstructive sleep apnea, children, sleep disorders, Taiwan

## Abstract

In this study, we collected and analyzed polysomnography (PSG) data to investigate the value of PSG in diagnosing sleep problems in children. The results of PSG studies of children (<18 years old) with sleep problems conducted from April 2015 to May 2017 at a children’s hospital in Taiwan were collected and analyzed retrospectively. Data for 310 patients (209 males and 101 females) who underwent PSG were collected. The final diagnoses were as follows: obstructive sleep apnea in 159 (51.3%), snoring in 81 (26.4%), limb movement sleep disorder in 25 (8.1%), hypersomnias in 12 (3.9%), central apnea in 8 (2.9%), enuresis in 7 (2.3%), bruxism in 5 (1.6%), sleep terrors in 5 (1.6%), narcolepsy in 3 (1.0%), sleep seizures in 3 (1.0%), sleep walking in 1 (0.3%), and insomnia in 1 (0.3%). PSG may help detect significant sleep-related problems in children and is useful for making therapeutic decisions regarding children. Obstructive sleep apnea syndrome (OSAS) was the primary sleep problem for most of the children (51.3%); however, only 7.4% of them underwent surgery for OSAS. We therefore suggest that children with sleep problems should undergo PSG.

## 1. Introduction

Sleep constitutes an opportunity for the body to conserve energy, restore its normal processes, promote physical growth, and support mental development. It also plays a vital and often underestimated role in the growth and development of children. Sleep problems have been reported to have high prevalence rates throughout childhood, affecting 25% to 50% of preschoolers and up to 40% of adolescents [1,2,3].

The taking of a detailed medical history is the first method that should be applied when screening for and identifying pediatric sleep problems, followed by polysomnography (PSG), which is a powerful diagnostic sleep medicine tool that can be used to record multiple different physiological parameters of a sleeping individual continuously and simultaneously.

In this study, we retrospectively collected the data of children who underwent PSG in our hospital due to sleep problems. The indications of these PSG studies and the final diagnoses of these patients were then analyzed and evaluated.

## 2. Materials and Methods

Ethical approval was provided by the ethical review board of China Medical University Hospital (CMUH103-REC2-082) on 9 July 2015. Patient consent was not required for this research. Data for children (<18 years) who presented to the outpatient department (OPD) of China Medical University Children’s Hospital for treatment of sleep issues and underwent PSG from April 2015 to May 2017 were collected and analyzed retrospectively. However, data for those who underwent follow-up PSG studies after adenotonsillectomy or for other reasons were excluded from this study.

The indications for PSG include snoring, sleep apnea, other sleep-related breathing disorders, excessive daytime sleepiness, limb movements while sleeping, bruxism, enuresis, sleep walking, insomnia, difficulty falling sleeping, and others.

A Nox Sleep Physiological Signals Recording System (Nox Medical ehf., Reykjavík, Iceland) was used to obtain PSG recordings, which were manually scored according to the American Academy of Sleep Medicine (AASM) scoring manual version 2.6. A level 1 PSG study is performed in our sleep laboratory with a sleep technologist present, and 12 channels are recorded, including 8 channels of electroencephalography (EEG), 2 channels of electrooculography (EOG), 1 channel of submentalis (chin) electromyography (EMG), and 1 channel of electrocardiogram (ECG)/heart rate, and 1 channel of pulse oximetry (SpO_2_). A multiple sleep latency test (MSLT) was also arranged for the day after any PSG study for those children who had a history of more than 3 months of excessive daytime sleepiness.

Obstructive sleep apnea syndrome (OSAS) was diagnosed based on the PSG data when obstructive events were noted and the apnea-hypopnea index (AHI) was one or more per hour, with 1 < AHI ≤ 5 taken to indicate mild OSA, 5 < AHI ≤ 10 taken to indicate moderate OSAS, and an AHI > 10 h of total sleep time (TST) taken to indicate severe OSAS.

The periodic limb movements (PLMs) during sleep were scored if there were at least four movements of 0.5–5 s duration that occurred between 5 s and 90 s apart. A PLM index of >5 per hour of sleep is generally considered to be rare in normal children, and therefore this threshold was used to define the presence of periodic limb movement disorder (PLMD).

Central sleep apnea (CSA) was defined as the absence of both inspiratory effort and chest wall movement lasting longer than 20 s when accompanied by a central apnea index greater than 1.

Furthermore, the presence of two or more sleep-onset rapid eye movement (REM) periods (SOREMPs) and a mean sleep latency of <8 min on the MSLT was regarded as being diagnostic of narcolepsy.

Categorical variables are presented as the frequency and proportion (%) and were analyzed by the chi-square test or Fisher’s exact test. All analyses were two-sided, and the significance level was set to 0.05. Statistical analyses were performed using SAS version 9.4 (SAS Institute Inc., Cary, NC, USA).

## 3. Results

Data for a total of 325 patients with sleep problems who came to the OPD for help from April 2015 to May 2017 and underwent PSG were collected in our study. Among these patients, a total of 242 patients had problems of snoring, sleep apnea, and other sleep-related breathing disorders. Forty of the patients had involuntary limb movements during sleep. Twenty-seven of the patients had symptoms suggestive of parasomnias such as bruxism, enuresis, and sleep walking. Twenty-two patients had a history of excessive daytime sleepiness, and one had difficulty falling asleep.

A total of 310 of the children (209 males and 101 females aged from 5 to 17 years old, with a mean age of 8.5 years and a median age of 10 years) had positive findings in the PSG and/or MSLT studies.

The final diagnoses of all patients are summarized in Table 1.

Among the 209 male patients included in this study, there were 5 male children who were diagnosed with both OSAS and PLMD. In the 109 male children with OSAS, 80 had mild OSAS, 24 had moderate OSAS, and 5 had severe OSAS. Furthermore, 27 of them had asthma or allergic rhinitis, 5 had obesity, 2 had Duchenne muscular dystrophy, 1 had prematurity, and 1 had mucopolysaccharidosis (MPS) type 2. In the 19 male children with PLMD, 4 had underlying diseases: 2 had developmental delays, 1 had Tourette syndrome, and 1 had hydrocephalus. In the six male children with central apnea, four had congenital heart disease after operation, one had ganglioglioma post operation, and the other did not have any underlying diseases.

Among the 101 female children included in this study, there were 2 female children who had both OSAS and PLMD. In 50 female children with OSAS, 36 had mild OSAS, 10 had moderate OSAS, and 4 had severe OSAS. Furthermore, 10 of them had allergic rhinitis, 2 had diabetes mellitus, 1 had Down syndrome, and 1 had Prader–Willi syndrome. In the 13 female children with PLMD, 4 had underlying diseases: 2 had a history of febrile convulsion, 1 had a history of syncope, and 1 had cerebral palsy. In the two female children with central apnea, one of them had congenital heart disease after operation, and the other did not have any underlying diseases. All of the patients’ final diagnoses are summarized in Table 1.

**Figure 1:**
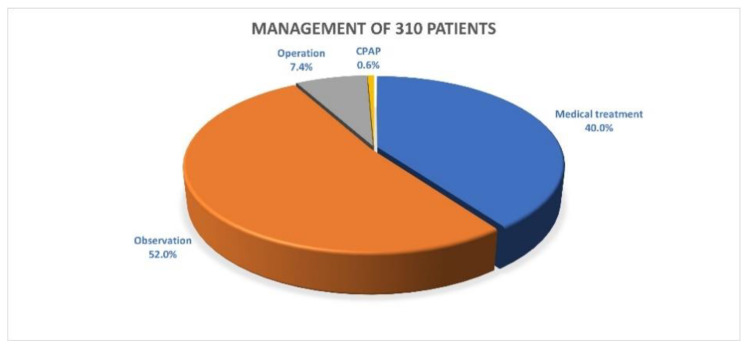
Management of 310 patients. CPAP = continuous positive airway pressure.

The methods used to manage 310 total patients with positive findings were as follows: operation with adenoidectomy and tonsillectomy was suggested for 37 patients with moderate or severe OSAS, and 23 patients (15 males and 8 females) (23/37 = 62%, or 7.4% of all the children enrolled in this study) underwent the operation; continuous positive airway pressure (CPAP) was used in 2 patients with central apnea (0.6%); medical treatment was used in 124 patients (40.0%); and the remaining patients were only placed under observation (*n* = 161, 52.0%) (Figure 1). For the 14 patients with moderate or severe OSAS who did not undergo an operation, a repeated PSG study 6–12 months later was suggested, but only 3 of them returned for follow-up visits, and their AHI results were decreased at those follow-ups.

## 4. Discussion

As with adults, there are all sorts of reasons why children do not sleep well. Some of those reasons are more serious than others. In our study, the sleep problem of the majority of the children (51.3%) was OSAS; however, only 7.4% of the total pediatric patients in the study underwent surgery for OSAS, even though some of the children with OSAS underwent surgery without undergoing preoperative PSG.

The etiologies of pediatric OSAS are multiple and can be classified into intrinsic upper airway narrowing or increased upper airway collapsibility [4]. Adenotonsillar hypertrophy is currently the most common cause of intrinsic upper airway narrowing, with other anatomical features resulting in upper airway narrowing, including craniofacial syndrome, achondroplasia, Down syndrome, Beckwith Wiedemann syndrome, and MPS. In our study, three patients had underlying diseases with intrinsic upper airway narrowing: one had Prader–Willi syndrome, one had Down syndrome, and one had MPS. Adenotonsillectomy was suggested for these three patients, but their families refused this surgical intervention.

A decrease in muscle tone in the upper airway can cause increased upper airway collapsibility, with the potential cause of such decreases including cerebral palsy, neuromuscular disorders, or inflammatory conditions such as allergic rhinitis and asthma. In our study, two OSAS patients had the underlying disease of Duchenne muscular dystrophy, and 24.5% (37/159) of the OSAS patients had allergic rhinitis or asthma.

Obesity appears to facilitate the development of OSAS; therefore, there is a high prevalence of OSAS among obese children. However, there is a higher proportion of children with OSAS who are obese. Thus, it appears that both OSAS and obesity can coexist and potentiate the adverse impacts of one another [5]. The prevalence of OSAS is increasing globally due to the growing occurrence of obesity in society. In obese children, the fat deposits in the upper respiratory tract cause breathing difficulties during sleep, thus causing OSAS [6,7], with reports of the prevalence of OSAS in obese children ranging from 13% to 59% [8]. In our study, 3.1% (5/159) of the children with OSAS were also obese, and all of them were male patients. One of these three patients had severe OSAS and underwent an adenotonsillectomy, while it was recommended that the other two patients achieve body weight reductions through an increase in their daily intense physical activities. A recent cross-sectional, prospective multicenter study, the NANOS study, assessed the contribution of obesity and adenotonsillar hypertrophy to pediatric OSAS and found that 46.6% of obese children in the community had OSAS [9].

Adenotonsillectomy is generally considered the first-line therapy in children with moderate or severe OSAS. In our study, there were 65 children with moderate OSAS and 9 with severe OSAS, and it was suggested that 26 of the moderate cases and all 9 of the severe cases undergo an operation. Of those 37 patients, 62% (23/37) underwent the operation. Some of the patients did not receive the operation because their family members wanted to further observe their symptoms for a period of time. For these fourteen patients who did not undergo the operation, repeated PSG studies were performed for three of them who come back for follow-up visits, and their AHI results were found to be decreased in those follow-ups.

PSG prior to adenotonsillectomy is indicated for children with some conditions that increase the risk of perioperative respiratory complications. These conditions include obesity (especially if severe), Down syndrome, craniofacial abnormalities, neuromuscular disorders, sickle cell disease, or MPS [10]. The purpose of the PSG in these high-risk children is to improve diagnostic accuracy and define the severity of OSAS to optimize perioperative planning. In our study, the OSAS children for whom the operation was suggested did have at least one of the aforementioned conditions, but some, including three with obesity, two with Duchenne muscular dystrophy, one with Down syndrome, one with MPS, and one with Prader–Willi syndrome did not undergo the operation.

Several authors have recommended that a clinical reevaluation be given to all children several months after adenotonsillectomy to determine whether snoring and the symptoms of OSAS had been resolved, especially in those children with higher risk of persistent disease, such as those with severe obesity or craniofacial syndromes. Furthermore, a postoperative PSG should be considered even in the absence of snoring or other symptoms in order to determine whether additional treatment is necessary for residual OSAS [4]. However, no studies to date have evaluated the timing of postoperative PSG evaluations, and this issue is not specifically addressed in the practice guidelines regarding the management of pediatric OSA. In our study, we did not collect postoperative PSG data, so further studies should be designed for evaluating this issue.

Medical therapies such as anti-inflammatory agents or CPAP are used as alternatives to adenotonsillectomy for children with OSAS, depending on the severity and specific locations of airway obstruction in the individual patient and on associated comorbidities.

The second most common sleep problem among the children in our study was primary snoring, and the management of pediatric primary snoring consists of treating any upper airway obstructions or observation in cases in which there is no upper airway obstruction.

The third most common sleep problem in our study was PLMD. A total of 31 children (10%) had PLMD proven by PSG, and 7 of those 31 also had OSAS. In the large clinical case series reported by Gingras et al. [11], PLMD was found to be common, affecting 14% of the 468 referred children. It is relevant to note that there are no Food and Drug Administration-approved treatments for PLMD in children. However, most children with PLMD have low iron storage; therefore, iron therapy should be considered as the first line of treatment in children with PLMD whose iron levels are low. In our study, the iron level was checked in nine children, and only one child with PLMD had a low iron level and was thus treated with iron therapy. Therefore, our management for PLMD consisted of non-pharmacologic treatments, such as education, massage, exercise, or observation only.

The fourth most common sleep problem in our study was idiopathic hypersomnia, which is extremely rare in children. Hypersomnia is present in 4% to 6% of the general population, with only 1% of the population having idiopathic hypersomnia and most of those people being adolescents or adults [12]. According to a report by Han et al. [13], 86% (361/417) of the children presenting with a complaint of primary hypersomnia to a sleep clinic in China met the criteria for narcolepsy with cataplexy, while only 20% (3/15) of the children with excessive daytime sleepiness in our study had narcolepsy.

The clinical characteristics and experiences of CSA are very limited in children compared with the adult population, and it is thought to occur in about 1–5% of healthy children [14]. CSA has been noted to occur more commonly in children with underlying diseases, and the presence of CSA may influence the course of those diseases. In our study, there were eight children who had CSA; six of them had secondary CSA, and two had idiopathic CSA. Idiopathic CSA is really rare in children, and it cannot be reliably identified or diagnosed on the basis of history or a specific set of signs and symptoms [14]. For the two children with a PSG-based diagnosis of CSA in this study, we suggested a magnetic resonance imaging (MRI) evaluation to assess for neuroanatomical abnormalities, but neither of the children came back for a follow-up visit.

A study by Felix et al. [15] reported that 18 of the 441 (4.1%) patients recorded during the study period had CSA, while 8 of the 310 (1.9%) patients in our study had central apnea. In the study, the underlying disorders were dominated by neurosurgical disorders; however, congenital heart diseases dominated in our study.

Many parasomnias in children can be recognized by history alone, but some require nocturnal PSG for appropriate diagnosis and management. In our study, there were 7 patients who had enuresis, 5 who had bruxism, and 1 who had sleep walking according to the PSG results, with these 13 patients accounting for 4.2% of all the patients who underwent PSG. We believe however that there are still so many children with parasomnias who do not go to pediatric OPDs for help.

PSG can be performed using either a level 1 laboratory PSG or a portable PSG. Some studies have attempted to evaluate the accuracy of portable PSGs in children, suggesting that a portable PSG may play an important role in the diagnosis of moderate and severe sleep apnea in older pediatric patients; however, a level 1 PSG remains the recommended method for diagnosis in children with sleep issues [16,17,18]. The disadvantages of a level 1 PSG include higher costs, limited availability, and the first night effect, which is characterized by decreased total sleep time, lower sleep efficiencies, reduction in REM (rapid eye movement) sleep, and longer REM latencies on the first night of testing [16,19].

There were several limitations in our study. The first was that our study was only a 2-year retrospective study, and some children with sleep problems, especially those suspected of having insomnias and parasomnias, may go to psychiatric OPDs for help, not pediatric OPDs, and thus may not undergo a PSG study. Second, long-term follow-up is necessary for children with sleep problems in order to observe whether their symptoms are relieved or their AHI results are decreased after treatment, especially for those with OSAS after adenotonsillectomy. Third, the first night effect may have been a confounding factor during our PSG study. Fourth, blood sample tests, such as tests of serum Fe or ferritin levels, are not typically conducted for children with PLMD. Further studies prospectively collecting PSG data from children with pediatric sleep disorders will thus be required.

## 5. Conclusions

The sleep problem of the majority of the children (51.3%) in this study was OSAS; however, only 7.4% of the total pediatric patients in the study underwent surgery for OSAS, even though some of the children with OSAS underwent surgery without undergoing PSG. We thus suggest that children with sleep problems should all undergo PSG studies, as PSG could help to detect significant sleep-related problems, and the application of PSG results is useful for making therapeutic decisions regarding children.

## Figures and Tables

**Table 1:** Final diagnoses of the patients and comparison between male and female patients. The values presented as n (%).

	Total (n = 310)	Male (n = 209)	Female (n = 101)	*p*-Value
OSAS	159 (51.3%) (7)	109 (52.2%) (5)	50 (49.5%) (2)	0.662
Primary Snoring	81 (26.1%)	57 (27.3%)	24 (23.8%)	0.510
PLMD	31 (10%) (7)	19 (9.1%) (5)	13 (12.9%) (2)	0.305
Idiopathic hypersomnia	12 (3.9 %)	8 (3.8%)	4 (4.0%)	1
Central apnea	8 (2.9%)	6 (2.9%)	2 (2.0%)	1
Enuresis	7 (2.3%)	6 (2.9%)	1 (1.0%)	0.434
Bruxism	5 (1.6%)	1 (0.5%)	4 (4.0%)	0.040
Sleep tremors	5 (1.6%)	3 (1.4%)	2 (2.0%)	0.662
Narcolepsy	3 (1.0%)	3 (1.4%)	0 (0%)	0.554
Sleep seizures	3 (1.0%)	1 (0.6%)	2 (2.0%)	0.249
Sleep walking	1 (0.3%)	0	1 (1.0%)	0.326
Insomnias	1 (0.3%)	1 (0.6%)	0	1

OSAS = obstructive sleep apnea syndrome; PLMD = periodic limb movement disorder.

## Data Availability

The data are not publicly available due to ethical restrictions.
